# A comparative study to assess synchronisation methods for combined simultaneous EEG and TMS acquisition

**DOI:** 10.1038/s41598-025-97225-7

**Published:** 2025-04-14

**Authors:** Isabela M. Miziara, Nicholas Fallon, Andrew Marshall, Heba Lakany

**Affiliations:** 1 Department of Musculoskeletal and Ageing, University of Liverpool, Liverpool, L7 8TX UK; 2 Technology Institute, Federal University of Pará, Belém, 66075-110 Brazil; 3 Department of Psychology, University of Liverpool, Liverpool, L69 3GF UK; 4 Pain Research Institute, University of Liverpool, Liverpool, L9 7AL UK; 5 Department of Clinical Neurophysiology, The Walton Centre NHS Foundation Trust, Liverpool, L9 7LJ UK; 6 Department of Electrical Engineering and Electronics, University of Liverpool, Liverpool, L69 3GJ UK

**Keywords:** Synchronisation, Transcranial magnetic stimulation, Electroencephalography, Lab Streaming Layer, Time inter-pulse error, Latency, Biomedical engineering, Electrical and electronic engineering, Data acquisition, Data processing

## Abstract

Electroencephalography (EEG) combined with transcranial magnetic stimulation (TMS) provides valuable insights into cortical excitability and connectivity but faces challenges including data artefacts, limited spatial resolution, and the need for standardised synchronisation protocols. This study evaluates three TMS-EEG synchronisation paradigms using the Lab Streaming Layer (LSL) to analyse time intervals and latency. Paradigm 1 employs a software-based approach with simultaneous pulses to both EEG and TMS devices. Paradigm 2, another software-based method, transmits a pulse to the TMS device first, followed by the EEG amplifier. Paradigm 3 uses a hardware-based approach where pulses generated by the TMS device are directly routed to the EEG amplifier. Synchronisation was assessed at frequencies of 1, 5, 10, and 20 Hz, with each frequency tested ten times using 100-pulse trains. Results demonstrate that Paradigm 3 provides superior performance, showing narrower distributions, lower time interval error (TIE) and latency values, and higher precision and accuracy. However, it requires a high sample rate from the EEG amplifier and limits additional device integration. Paradigms 1 and 2, while exhibiting greater variability and lower precision, allow for additional device integration and inter-pulse control via LSL. All paradigms achieved low latency and timing error values within acceptable limits for EEG applications, affirming their viability. The choice of synchronisation paradigm has a significant impact on performance, and the current lack of standardisation in TMS-EEG studies presents ongoing challenges. These findings underscore the necessity of selecting an appropriate synchronisation method based on specific study requirements and resources, potentially advancing standardised protocols for TMS and enhancing the reliability of TMS-EEG research.

## Introduction

Transcranial magnetic stimulation (TMS) is a non-invasive technique that uses a strong, brief magnetic field to excite axons in the brain and depolarise them effectively, initiating action potential propagation^1^. Trans-synaptic activation of neurons’ excited axons leads to arising postsynaptic currents in the dendrites, which may undergo spatial and/or temporal summation, reach a significant level, can be recorded by different techniques such as EEG, functional magnetic resonance imaging (fMRI), near-infrared spectroscopy and positron emission tomography^2–4^. Among these techniques, the most successful and thus commonly used combination has been with EEG because it is a widespread method, more affordable than other neuroimaging techniques, and is technically the least complicated to be combined in real-time with TMS^5^.

The combination of TMS and EEG (TMS-EEG) offers a powerful technique for probing cortical excitability, reactivity, connectivity, the instantaneous state, and modulating the brain^5,6^. In brain connectivity evaluation, TMS-EEG offers several advantages. It provides a neurophysiological marker of excitability or connectivity for any brain area^7^ and can be combined with other neuroimaging techniques, such as fMRI, to improve spatial resolution^8^.

The first effort to combine TMS with EEG was reported in 1989 by Cracco^9^ and later by Amassian^10^. In 1996, the first successful TMS-EEG study^7^ demonstrated the feasibility of recording cortical excitability and connectivity using this combination. After these first successful recordings, the interest in using EEG to measure brain activation elicited by TMS has steadily increased. Despite, more than three decades since the first TMS-EEG study, no consensus remains on standardised procedures for TMS-EEG preparation, data acquisition, or analysis^5^.

While TMS-EEG is a valuable tool for investigating brain function^5,11–15^, it presents several limitations. A primary challenge is the presence of artefacts in the EEG data induced by the TMS pulse including electromagnetic interference, electrode movement, muscle activation, and auditory evoked potentials from the coil click)^6,16,17^, which can compromise data quality and impact result accuracy. Moreover, single-trial variability and noise in TMS-EEG recordings^18^ further complicate result interpretation. The lack of standardised protocols for TMS-EEG synchronisation^19^ hinders cross-study comparisons.

Precise multimodal data alignment is crucial to ensure consistent temporal properties across TMS-EEG trials. Synchronising TMS pulses with ongoing EEG activity offers several key advantages, including reducing trial-to-trial variability in TMS-evoked EEG artefact and the identification of recovery times for the immediate physiological response in the stimulated cortex and connected areas^18^. Accurate synchronisation of TMS-EEG data enables the study of how TMS influences the oscillatory state of the targeted brain region^11^ and the phase dynamics associated with changes in excitability. This insight may enhance the effects of TMS and improve the efficacy of stimulation protocols^20^. Additionally, TMS-EEG synchronisation facilitates investigation of the causal roles of specific oscillatory frequencies and phases in cognitive functions at specific moments^11,21^, as well as the propagating of TMS effects across brain networks over time, i.e., functional connectivity, by analysing TMS-evoked potentials (TEPs) in EEG signals^12^.

Real-time EEG-TMS integration enables the development of adaptive systems that detect and process ongoing brain signals to deliver TMS stimuli at targeted oscillatory phases^8^. This adaptive capability allows for neurofeedback and rapid, phase-locked adjustments to TMS parameters. Previous studies have advanced this capability by developing an automated closed-loop TMS-EEG setup that electronically adjusts stimulus parameters via multi-locus TMS. As a proof of concept, an algorithm was implemented to optimise stimulation orientation based on single-trial EEG responses, achieving the electric field orientation that maximises TMS-EEG response amplitude^22^. Such advancements in real-time modulation have shown promise in enhancing prefrontal excitability to support working memory, as evidenced in studies using EEG-triggered theta-phase rTMS synchronised with the left dorsomedial prefrontal cortex^23^.

Proper synchronisation of EEG recordings with external events or other data streams is complex. Failure can have severe consequences, rendering the data unusable and leading to incorrect conclusions. The main implications of inadequate synchronisation are inaccurate event-related potential (ERP) analysis, an inability to establish precise temporal relationships, misalignment of multimodal data, failure of real-time applications, and data corruption and quality issues. The register delays are highly relevant in neurofeedback applications because the delays should be under 100 ms to achieve real-time adjustments and optimise TMS treatment individually^24^. Thus, it is necessary to discuss the impact of inadequate data synchronisation on the neurophysiological analysis of multimodal datasets in EEG and TMS studies. The various recording parameters of each device, such as sampling rate, data transfer protocols, artefacts, software, and hardware, substantially influence multimodal measurements and synchronisation. Furthermore, the temporal variance of data acquisition, as a consequence of the heterogeneity of device settings in TMS-EEG experiments, necessitates additional costs for data harmonisation^25^.

Commercial research device manufacturers provide communication protocols for device integration; however, these protocols vary widely. Several methods have been employed to synchronise EEG signals with other devices, including auxiliary channels integrated into the EEG amplifier, hardware-based triggers generated by external devices, and software-based synchronisation using communication protocols to transmit data packets via local networks. External devices, such as TMS, are frequently used in studies of neural processing related to specific tasks, where precise time alignment is largely dependent on the availability of hardware-based triggers, such as transistor-transistor logic (TTL), serial communication, or analogue output^26–29^. Generally, hardware-based synchronisation is reliable enough for behavioural and electrophysiological studies. The most common communication method is direct BNC connections, which, while simple, have limitations. These include the inability to precisely measure system delays and jitters, lack of flexibility for complex experimental designs, unmeasured variability between setups, and restricted use for basic trigger-based synchronisation between two devices. Additionally, they do not support real-time data streaming or more complex multi-device configurations.

Developing multi-device systems with advanced synchronisation capabilities is essential for studies that investigate complex neural dynamics, such as those examining phase-dependent modulation of cortical signal transmission through combined use of transcranial alternating current stimulation (tACS), TMS, and EEG^30^. In such studies, precise synchronisation across multiple devices is crucial to explore the causal role of neural oscillations in cortico-cortical communication. This type of multi-device setup not only enables real-time monitoring and manipulation of neural oscillations but also underscores the necessity of synchronisation protocols that extend beyond simple trigger-based connections. Advanced multi-device configurations that allow for real-time data streaming and intricate timing arrangements would provide a robust framework to dynamically investigate brain networks. By enhancing multi-device integration, future studies could more effectively probe the mechanistic basis of neural signal transmission, yielding valuable insights that single-device setups cannot achieve.

An alternative approach for the time alignment of signals from multiple devices is software-based or assisted synchronisation. These approaches align the acquired signals using a common time series with the computer clock as a reference. Some human neuroscience studies already use platforms that facilitate temporal alignment, e.g., the Lab Streaming Layer (LSL)^31^. The LSL facilitates the synchronisation of multimodal and EEG signals through real-time data streaming, processing, and integration, eliminating the need for hardware triggers^25,31^. This tool provides a flexible framework for managing multiple devices within a local network. LSL allows additional devices by implementing sub-modules based on provided libraries. The LSL network collects signals from connected client computers or virtual devices, microsecond-precision timestamping, and automatically aligns them through the central recording system, LabRecorder. Due to its functionalities, the LSL tool supports various real-time processing pipelines and feedback mechanisms.

This study addresses the critical challenges of TMS-EEG synchronisation by focusing on measuring and optimising system delays, which is essential for real-time applications. The study aims to develop and evaluate flexible and precise synchronisation methods that accommodate multiple systems and allow accurate time interval and latency measurement. We propose various TMS-EEG synchronisation paradigms and assess their effectiveness by analysing the time intervals between pulses in a pulse train typical of rTMS applications and the latency between connected devices. This study utilises the LSL tool to enhance synchronisation accuracy and evaluation. This tool enables real-time data streaming, processing, and integrating multiple devices.

This study compared three TMS-EEG synchronisation paradigms encompassing hardware and software-based approaches. LSL records precise timestamp data, facilitating a thorough evaluation of synchronisation accuracy and allowing for dynamic adjustment of TMS parameters based on ongoing EEG activity. Additionally, we detailed hardware and software specifications, methodologies for measuring and optimising performance, and benchmarks for comparing different configurations.

Besides that, this study aims to offer a replicable solution for TMS-EEG integration, focusing on precise quantification of system delays and jitters and support for advanced real-time applications. By examining the advantages and disadvantages of each synchronisation method, we aim to contribute to the development of standardised protocols and assist researchers in selecting the most appropriate synchronisation methods for their specific study requirements. The insights gained will be valuable for labs seeking to implement precise, flexible, and well-characterised TMS-EEG setups, regardless of their hardware configurations.

## Results

The results presented in this study focus on the time interval and latency analysis, both derived from data collected from each virtual device across three evaluated paradigms. Paradigm 1 is a software-based synchronisation method developed to send computer-generated pulses simultaneously to both the EEG amplifier and the TMS device. Paradigm 2 is another software-based synchronisation method that generates a computer pulse and sends it to the TMS device and then to the EEG amplifier. Paradigm 3 is a hardware-based method where pulses are generated by the TMS device and delivered to the EEG amplifier. The recorded pulse timestamps for all paradigms reflect the precise timing alignment of TMS pulses with EEG signal acquisition, ensuring synchronisation of the devices.

Timestamp recordings for each paradigm were assessed under four distinct setups of pulse train frequencies: 1, 5, 10, and 20 Hz. Each setup was replicated ten times, with each trial comprising a train of 100 pulses. Data were recorded from the following virtual devices: App, Markers, EEG, and TMS. However, the Markers virtual device was excluded from the analysis due to data ambiguity with the EEG virtual device and the recording discrepancies. Consequently, event marker data were extracted from one of the 33 channels of the EEG time series. The mean values and standard deviations of time interval and latency analysis are available in table format in Supplementary Files.

### Time interval analysis

Pulse intervals between pulses (timestamps) were calculated to analyse the time interval data. The difference between the observed and expected intervals, based on the adopted frequency, was computed to standardise the data across different pulse train frequencies. This difference, termed the Time Interval Error (TIE), was then used to generate histograms, normal distribution curves, and box plots.

To further interpret the data, statistical hypothesis testing was performed using the non-parametric Kruskal–Wallis test, as the data did not follow a normal distribution according to the Kolmogorov–Smirnov test. The null hypothesis (H0) assumed no significant statistical differences between variations, while the alternative hypothesis (H1) posited that significant differences existed. Significant p-values (p < 0.05) and corresponding comparisons are highlighted in the figures. Additional p-values, including non-significant comparison results, and detailed results from the statistical tests are provided in Supplementary Files.

**Fig. 1:**
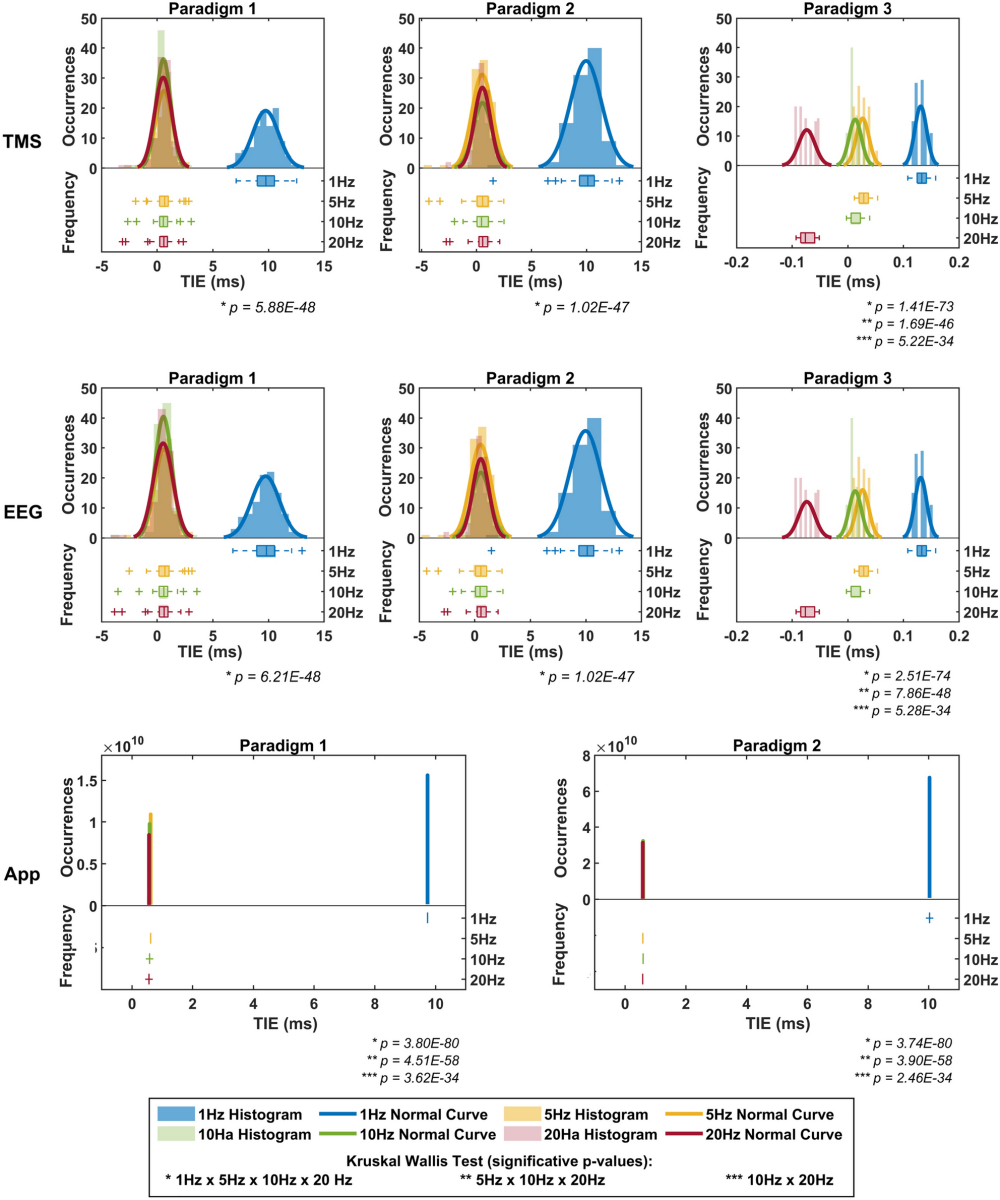
TIE (in milliseconds) across different paradigms for each virtual device. Significant *p-values* from the Kruskal–Wallis statistical test are indicated below each graph, where comparisons with $\textit{p < 0.05}$ are denoted by asterisks (*), as detailed in the figure legend. The App device, displayed as a straight line at the mean TIE, shows only the normal curve due to the lack of variation in the computer-generated pulse intervals. TMS and EEG Paradigms 1 and 2 are shown on the same time scale, while Paradigm 3, having substantially different values, is plotted on a separate time scale. The App results are presented using different scales for both time and magnitude to enhance visual clarity.

Figure 1 displays histograms, normal curves, and box plots of TIE in milliseconds, derived from timestamp data recorded by each virtual device (EEG, TMS, and App) across frequency setups (1, 5, 10, and 20 Hz) for the applied paradigms. Data from the EEG and TMS devices were collected for all three paradigms, while the App device was recorded for Paradigms 1 and 2. Significant p-values and corresponding comparisons are indicated in the figure.

The TMS and EEG virtual devices exhibited greater mean TIE values and a distribution offset in the histogram under a 1 Hz frequency setup across all paradigms. In Paradigms 1 and 2, the TIE histograms showed a wider distribution compared to Paradigm 3, which displayed a narrower TIE distribution. Paradigm 3 presented distinct TIE values and distribution ranges for the different frequency setups, except for the 5 Hz and 10 Hz frequencies, which had similar TIE values and overlapping histograms. For the TMS TIE results, except for the 1 Hz frequency setup in Paradigms 1 and 2, the TIE values were close to zero.

The TIE of the App virtual device remained constant throughout the pulse train for all four evaluated frequencies, as the pulse trains were computer-generated. Consequently, the distribution of occurrences could not be analysed using histograms and box plots, and the normal curve is represented as a straight line at the TIE mean value. Both paradigms showed TIE values between 0 and 1 ms for setups at 5, 10, and 20 Hz and close to 10 ms for the 1 Hz setup.

No statistically significant differences were found for the same parameters across TMS, EEG, and App devices when comparing the virtual devices. However, significant differences emerged when comparing TIE values across different frequency setups, with the 1 Hz configuration being statistically different from the other frequencies.

**Fig. 2:**
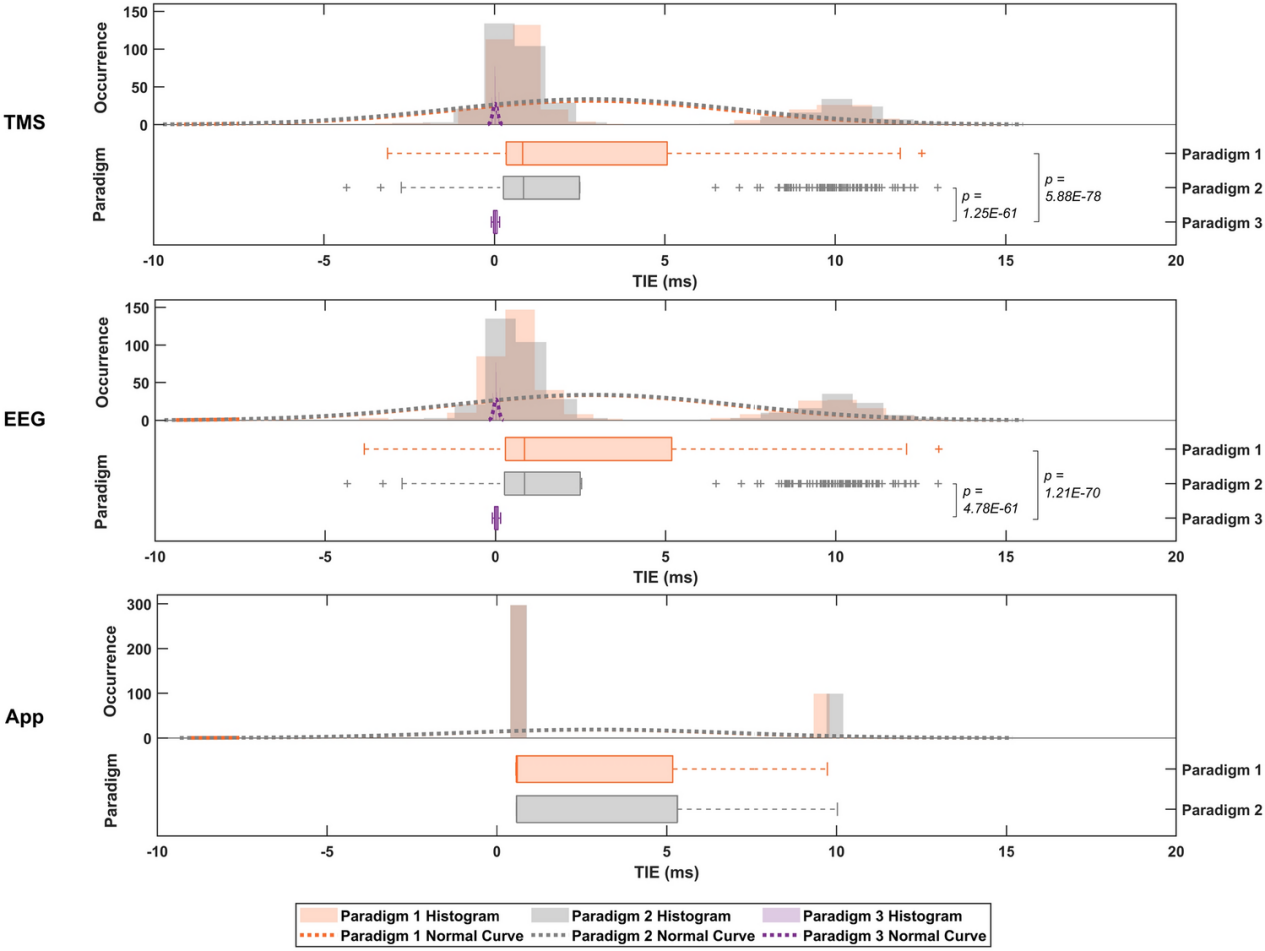
TIE (in milliseconds) values across paradigms on a unified time scale and grouped by virtual device. Significant *p-values* ($\textit{p < 0.05}$) from the Kruskal–Wallis test are marked on the graph, with corresponding comparisons identified.

Figure 2 provides a comprehensive overview representing, in the same graph, the TIE distribution obtained from the three paradigms across all four frequency setups for each virtual device. The data are displayed on the same scale to facilitate a comparison of TIE across different virtual devices under these paradigms. Significant p-values and corresponding comparisons are indicated in the figure.

According to the results displayed in Fig. 2, despite the TIE values from the TMS and EEG virtual devices being similar across Paradigms 1 and 2, Paradigm 1 exhibited a broader TIE distribution, while Paradigm 2 presented numerous outliers. In contrast, Paradigm 3 demonstrated a narrower distribution and lower TIE values, as seen in Fig. 1. Paradigms 1 and 2 showed a broader TIE distribution for the App virtual device due to the discrepant values obtained among the frequency setups, particularly at 1 Hz. Despite the extensive App box plots, it is essential to emphasise that the App TIE values did not exhibit variability due to the computer-generated pulses, as observed in Fig. 1.

Statistical analysis of TMS TIE values across paradigms revealed that Paradigm 3 had a significant *p-value* (< 0.05) compared to other paradigms, leading to the rejection of the null hypothesis and indicating that Paradigm 3 is statistically different from the others at a 95% confidence level. Similar results were found for EEG TIE values. However, no significant differences were detected in the TIE values of the App device between Paradigms 1 and 2.

The analysis of IPI Relative Error and TIE Relative Uncertainty (both in percentage) is summarised in Table 1. The results are organised by virtual device, each evaluated across three paradigms and four frequency setups. Table 1 shows varying relative uncertainty values at the 1Hz setup. A higher IPI relative error was observed for all virtual devices at 20 Hz across all paradigms, with Paradigms 1 and 2 showing greater error than Paradigm 3. Additionally, higher relative uncertainty was found in Paradigms 1 and 2 for both TMS and EEG devices. Specifically, Paradigm 3 showed lower uncertainty at 1 Hz and 20 Hz, higher uncertainty at 10 Hz, and the smallest IPI relative error overall. For the App virtual device, consistently low relative uncertainty was observed across all frequency setups, likely due to the low variability in computer-generated pulses. The App device was not evaluated for Paradigm 3.

**Table 1:** IPI relative error and TIE relative uncertainty.

Virtual device	Paradigm	Frequency (Hz)	IPI relative error (%)	TIE relative uncertainty (%)
TMS	Paradigm 1	1	0.973	11.887
		5	0.306	122.589
		10	0.565	117.299
		20	1.078	143.154
	Paradigm 2	1	0.995	14.444
		5	0.262	176.237
		10	0.553	150.980
		20	1.091	134.808
	Paradigm 3	1	0.013	10.134
		5	0.013	56.088
		10	0.013	92.925
		20	0.147	14.783
EEG	Paradigm 1	1	0.972	12.838
		5	0.305	135.940
		10	0.564	134.749
		20	1.075	165.416
	Paradigm 2	1	0.995	14.470
		5	0.262	176.5667
		10	0.553	150.958
		20	1.089	134.964
	Paradigm 3	1	0.013	8.098
		5	0.013	43.196
		10	0.013	85.314
		20	0.148	19.990
App	Paradigm 1	1	0.973	2.611E−9
		5	0.310	5.816E−8
		10	0.586	6.905E−8
		20	1.133	8.254E−8
	Paradigm 2	1	1.003	5.824E−10
		5	0.291	2.434E−8
		10	0.591	2.066E−8
		20	1.157	2.178E−8

### Latency analysis

Latency generally refers to the time delay between an action and its corresponding effect or response in various systems. In this context, for this study, the latency measurements were obtained between TMS and EEG (TMS-EEG) across all three paradigms and between App and TMS (App-TMS) and App and EEG (App-EEG) across Paradigms 1 and 2.

As in the TIE analysis, the non-parametric Kruskal–Wallis test was applied for latency between virtual devices, following confirmation of non-normal data distribution using the Kolmogorov–Smirnov test. Latency results were statistically compared across all paradigms for each device. Significant p-values (p < 0.05) and corresponding comparisons are highlighted in the figures, with additional p-values and detailed statistical results provided in the Supplementary.

**Fig. 3:**
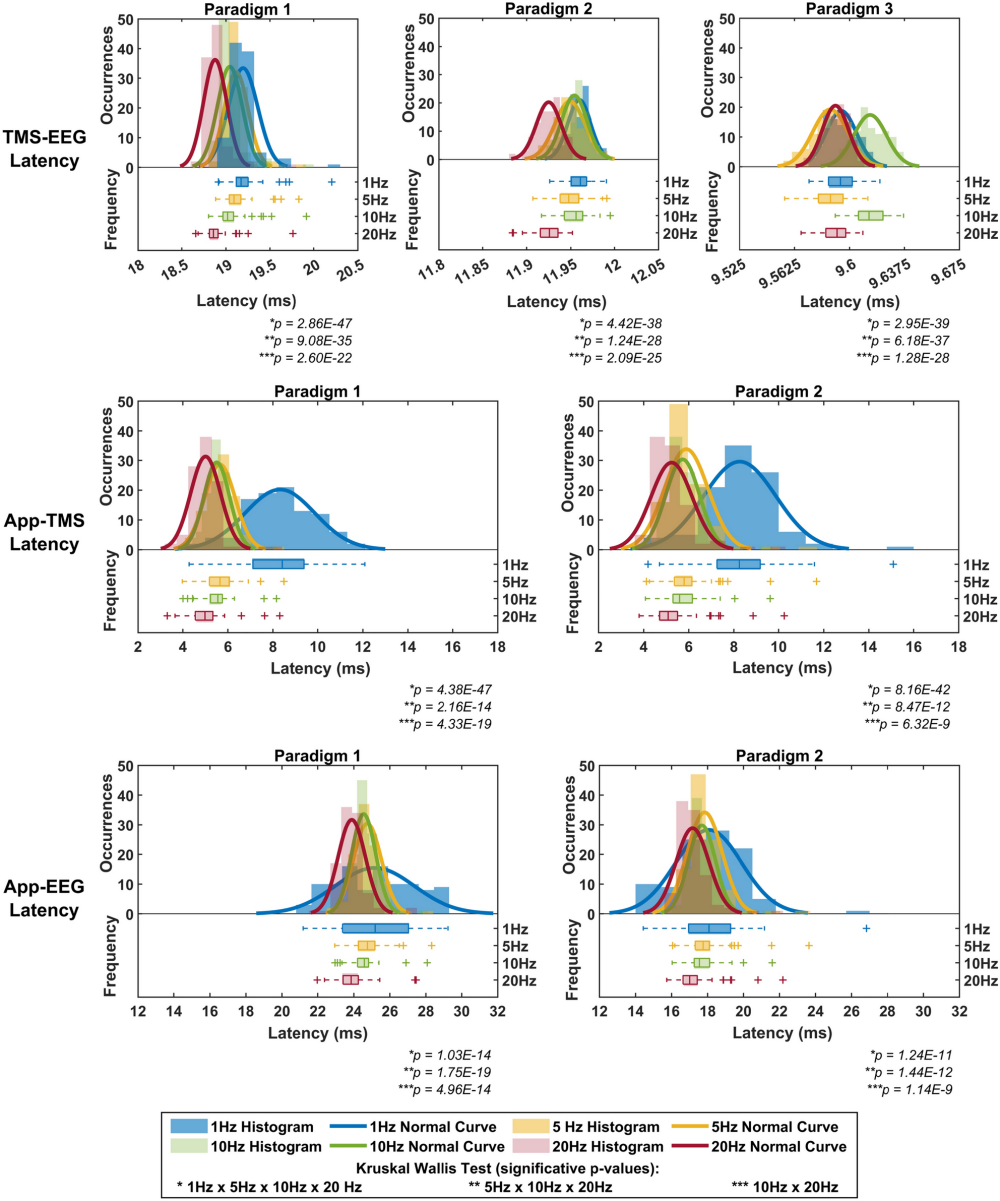
Latency (in milliseconds) measured between the virtual device timestamps for each analysed paradigm. Significant *p-values* from the Kruskal–Wallis test are shown below each graph, with comparisons where $\textit{p < 0.05}$ marked by asterisks (*), as detailed in the legend. While all graphs use the same magnitude scale, varying time scales were applied to enhance the visualisation of the distributions.

Figure 3 illustrates the histograms, normal curves, and box plots of the latency (in milliseconds) measured between the virtual device’s timestamps data recorded by each virtual device (EEG, TMS, and App) across frequency setups (1, 5, 10, and 20 Hz) for the applied paradigms. Significant p-values and corresponding comparisons are indicated in the figure.

Statistical tests confirmed significant differences in latency across all frequency configurations and paradigms. All paradigms showed latency values different from zero, with distinct distributions, variances, and mean values. Paradigm 1 exhibited the highest latency variability for TMS-EEG latency, while Paradigm 3 showed the least. Regarding latency distribution, Paradigms 1 and 2 had the highest mean latency at 1 Hz and the lowest at 20 Hz, whereas Paradigm 3 had the highest mean latency at 10 Hz. The latency values of the App-EEG and App-TMS configurations were generally higher. Paradigms 1 and 2 displayed substantial variability and a broad distribution of values at 1 Hz, with the lowest mean latency observed at 20 Hz.

**Fig. 4:**
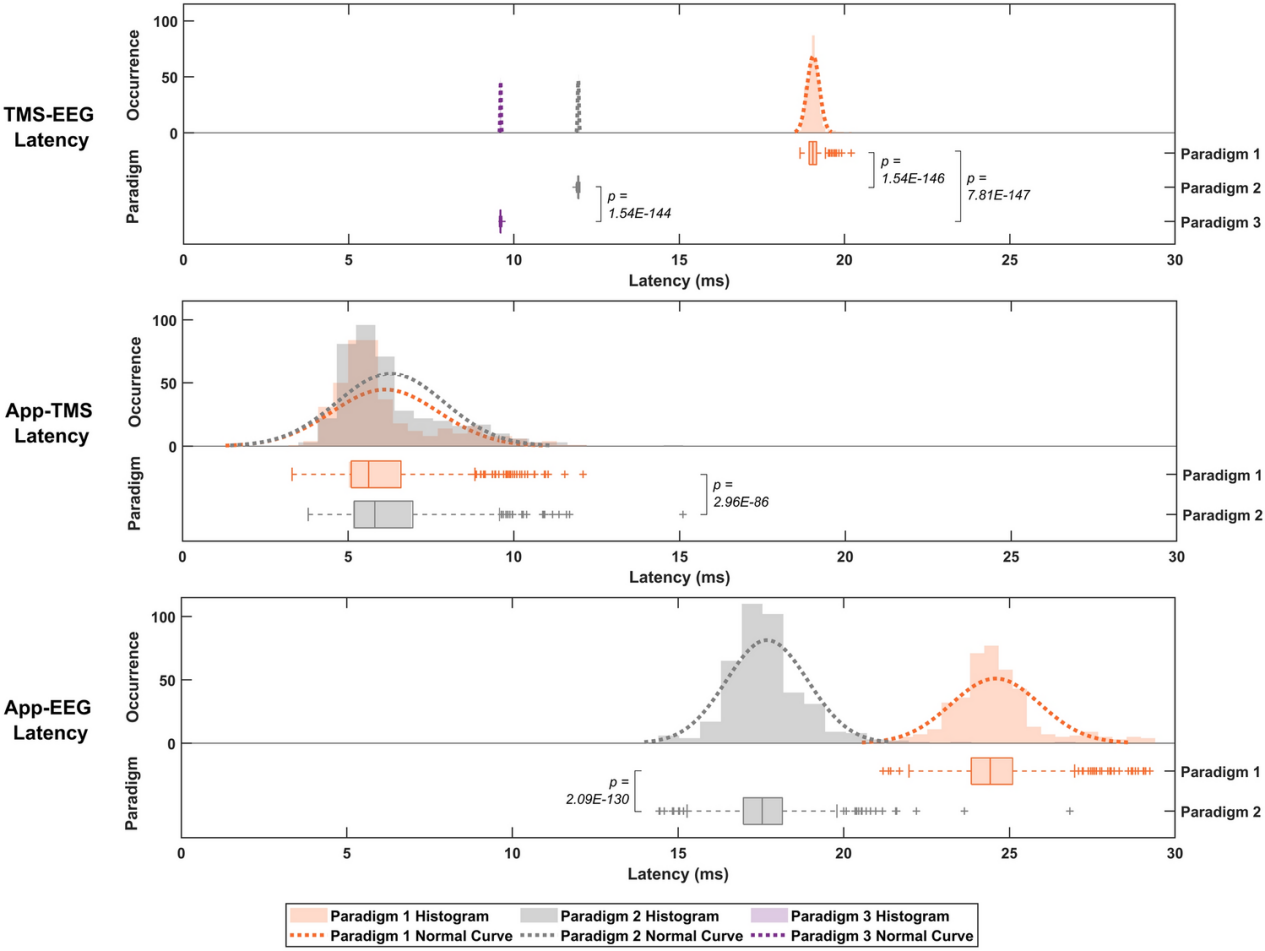
Latency values (in milliseconds) are presented across paradigms on a unified time scale and grouped by virtual device. Significant *p-values* ($\textit{p < 0.05}$) from the Kruskal–Wallis test are highlighted on the graph, with comparisons indicated.

Figure 4 provides a comprehensive overview of latency behaviours across different paradigms, highlighting the variations and similarities in latency distributions and mean values. To compare the behaviour across different paradigms, Fig. 4 presents the distribution of all latency values for each paradigm on the same scale, considering the total values obtained for all frequency setups. To analyse TMS-EEG, App-TMS, and App-EEG latency, histograms, normal curves, and box plots were employed to illustrate the data for all adopted paradigms. Significant p-values and corresponding comparisons are indicated in the figure.

Figure 4 shows that TMS-EEG latency results exhibit distinct distributions across paradigms. Paradigms 2 and 3 have narrower distributions and shorter latency ranges compared to Paradigm 1, consistent with the findings in Fig. 3. Paradigm 3 demonstrates the lowest mean latency, whereas Paradigm 1 shows the highest mean latency. The distributions between the two analysed paradigms are similar for App-TMS latency, with overlapping histograms in Fig. 4. Despite this similarity, Paradigm 2 shows higher mean latency due to outliers, making Paradigm 1’s mean latency lower. In contrast, App-EEG latency reveal more distinct distributions. Paradigm 1 has the highest mean latency and a distribution skewed towards higher latency values compared to Paradigm 2. Unlike the App-TMS analysis, where histograms of the two paradigms overlap substantially, the App-EEG analysis shows minimal overlap. The Kruskal–Wallis test confirmed significant differences across all paradigms for each latency analysis. Comparing all three latency analyses, App-TMS latency, though variable, are generally lower than other paradigms. TMS-EEG Paradigms 3 and 2 show lower latency with minimal variation, followed by App-EEG Paradigm 2, TMS-EEG Paradigm 1, and App-EEG Paradigm 1.

Table 2 summarises the Relative Uncertainty of latency values across the virtual devices. All paradigms showed low relative uncertainty for the TMS-EEG latency analysis, with Paradigm 1 exhibiting the highest values. In contrast, the App-TMS latency results displayed greater relative uncertainty compared to other analyses across all paradigms, with higher means and accuracy observed at 1 Hz. The App-EEG latency results showed intermediate relative uncertainty, also with higher values at 1 Hz, similar to the App-TMS results.

**Table 2:** Latency relative uncertainty.

Latency	Paradigm	Frequency (Hz)	Relative uncertainty (%)
TMS-EEG	Paradigm 1	1	0.871
		5	0.715
		10	0.742
		20	0.700
	Paradigm 2	1	0.109
		5	0.143
		10	0.121
		20	0.119
	Paradigm 3	1	0.108
		5	0.125
		10	0.117
		20	0.093
App-TMS	Paradigm 1	1	18.759
		5	12.096
		10	10.375
		20	13.230
	Paradigm 2	1	19.569
		5	16.447
		10	13.084
		20	17.441
App-EEG	Paradigm 1	1	8.727
		5	3.124
		10	2.706
		20	3.159
	Paradigm 2	1	10.171
		5	5.429
		10	4.238
		20	5.326

## Discussion

This study analysed three synchronisation paradigms to evaluate their performance in simultaneous data acquisition using TMS and EEG devices. Paradigm 1 offers some advantages that could make it appealing for certain research scenarios. Using an external computer to communicate with the TMS device provides flexibility and easy integration with other devices or research tools. Paradigm 1 setup also benefits from a lower sampling rate for recording EEG data. It reduces computational load and data storage requirements and makes managing and analysing data easier, particularly in long-duration studies in real-time. In addition, Because it is a software-based synchronisation method, through the use of tools such as LSL, it is possible to precisely measure and optimise all system delays, which is crucial for true real-time applications. Compatibility with other devices and a simplified app interface contribute to the usability of this configuration, especially for researchers who may not have extensive technical training.

Paradigm 2 retains several advantages of Paradigm 1, including using an external computer for communication, compatibility with other devices, timestamp delay measurement via LSL, and a simplified application interface. These features contribute to greater flexibility, ease of use, faster setup times. However, there are notable limitations despite the simplicity of using BNC connections in this paradigm for direct communication between EEG and TMS. These include variability between setups and the requirement for a high sampling rate from the EEG amplifier to detect the short electrical pulse from the TMS accurately. These constraints increase computational and data storage demands, potentially posing challenges in research environments with constrained resources.

One of the key advantages of Paradigm 3 is the integration of the TMS app, a dedicated control interface that facilitates setup and pulse delivery. Notably, the app allows for the direct determination of the motor threshold, potentially improving the accuracy of threshold integration in specific stimulation protocols^32^. In addition to its user-friendly design, which minimises operator error and simplifies manipulation, the TMS app offers a wide range of protocol settings and patient registration options, making it highly suitable for clinical applications. However, Paradigm 3, similar to Paradigm 2, relies on hardware-dependent synchronisation, requiring a high sampling rate for precise EEG data acquisition. This introduces challenges, including unaccounted-for variability across setups and the inability to accurately measure system delays and jitters throughout all communication stages. Furthermore, its performance is contingent on the hardware specifications, complicating the integration of multiple devices and signals. These issues pose significant challenges in data management, real-time applications, and computational resource allocation.

For the quantitative evaluation of this study, LSL technology was employed to record timestamp data from three virtual devices within the synchronisation system: the App, TMS, and EEG devices. Each device recorded timestamps using the LSL-integrated tool, LabRecorder. The collected data were subsequently processed to determine the inter-pulse interval (IPI), defined as the time difference between two consecutive pulses; the timing interval error (TIE), defined as the difference between the received and expected timestamps; and latency, defined as the time interval or delay between the respective timestamps received by two different virtual devices.

The results obtained for TIE values from the virtual devices analysed demonstrated similar behaviour for paradigms 1 and 2, as evidenced by the lack of statistically significant differences, including the App paradigms, which, despite the absence of variations, the TIE results showed similar mean values from the other devices. The absence of differences between the software-based paradigms indicates that delivering the pulse simultaneously to both TMS and EEG, as well as sequentially from TMS to EEG, did not impact the TIE values. However, Paradigm 3 of the TMS and EEG virtual devices exhibited significant differences and distinct behaviour compared to the other paradigms, like smaller variations and negative TIE values at the 20 Hz setup. Although unexpected, negative TIE values are common and can occur in network measurements and signal analysis, indicating that a packet or signal edge has arrived earlier than expected^33^. Although negative TIE may seem beneficial, it can cause system problems, leading to buffer starvation if not appropriately managed^33^.

Moreover, a statistical difference was observed in the 1 Hz frequency setup data across all virtual devices and paradigms due to higher mean TIE values and distribution displacement. This behaviour is a consequence of the delay in delivery or the computer recording of pulses, as corroborated by the TIE results of the App device. Additionally, low-frequency signals are susceptible to noise, which can cause early or late triggering, thus affecting TIE measurements. High-frequency noise can also impact TIE by causing jitter in the measurements. Extended observation periods are required to capture slow variations or wander in low-frequency signals. This helps in accurately characterising the long-term stability of the oscillator^34^. Thus, a low-frequency signal TIE can substantially affect digital systems. It is often more problematic than a high-frequency TIE as it accumulates over time, leading to more significant timing errors. This accumulated TIE, also represented by the jitter phase, causes the timing edges to be significantly displaced from their ideal locations^35^.

Regarding latency, the results revealed statistically significant differences between TMS-EEG and App-EEG analysis paradigms. Nevertheless, these differences are attributed to the synchronisation methods adopted by each paradigm. For Paradigm 1, lower latency was expected, as the pulse is generated and delivered simultaneously from the App to the TMS and EEG despite possibly experiencing registration delays. In Paradigm 2, the App generates the pulse, which is delivered to the TMS and subsequently to EEG, resulting in more significant latency between App-EEG, as illustrated in Fig. 4. In Paradigm 3, the TMS system generates the pulse and then delivers it to the EEG, resulting in the lowest latency because the App does not mediate it. On the other hand, the latency between the App and TMS did not show significant differences between Paradigms 1 and 2, indicating that, regardless of the synchronisation method used, the time intervals remained consistent, and the use of additional virtual devices did not interfere with pulse delivery times.

Although LSL has very low latency, even over a network, the absolute latency depends on the devices involved in the synchronisation due to factors such as hardware buffer filling or USB transmission, which cannot be changed. Factors such as operating system, drivers, and hardware performance will also introduce latency variations in real systems^36,37^.

The precision and accuracy of the timestamp registration are also crucial factors to consider. Accuracy refers to how close a measurement is to the true or accepted value, indicating minimal systematic error or bias. Precision, however, pertains to the consistency of results, regardless of their closeness to the true value. This study investigated the correlation between the precision and accuracy of time interval analysis results using two specific metrics: TIE relative uncertainty and IPI relative error. An increase in TIE relative uncertainty indicates a reduction in precision, while an increase in IPI relative error is inversely related to accuracy. For the latency analysis, the precision is inversely correlated with the relative uncertainty of the results.

When analysing the time intervals, similar precision results were observed for both the virtual EEG and TMS devices across all paradigms, with precision inversely proportional to relative uncertainty. Precision was highest for configurations using a frequency of 1 Hz, as expected, due to statistically significant differences in time intervals at low frequencies, reduced electromagnetic interference, and cleaner signals. This aligns with previous studies showing that jitter above 10 ms significantly decreases the signal-to-noise ratio in visual evoked potentials^25^. In Paradigm 3, higher precision was also noted at 20 Hz.

Regarding accuracy, the low relative error in the IPI demonstrated good accuracy across paradigms, though slightly reduced for 1 and 20 Hz configurations. The virtual device App provided accurate data with low relative uncertainty values, particularly at 1 and 20 Hz for all three paradigms.

App-TMS latency demonstrated the lowest precision among the latency analysed in this study, while Paradigm 3 exhibited the highest precision values among the TMS-EEG latency paradigms. Specifically, for latency analysis, the 1 Hz frequency setup yielded reduced precision, particularly affecting the App device in the context of both App-TMS and App-EEG latency.

Despite the different configurations between the synchronisation methods adopted, Paradigms 1 and 2 demonstrated similar behaviours. Conversely, Paradigm 3 exhibited distinct behaviour, characterised by lower levels of variation and lower mean TIE values. Additionally, Paradigm 3 presented lower latency values between the primary devices focused on in this study, TMS and EEG. Paradigm 3 also showed low relative uncertainty and relative error for inter-pulse values in time interval analysis, as well as low relative uncertainty in latency analysis, making this paradigm more precise and accurate. This characterises Paradigm 3 as more advantageous. However, Paradigm 3’s synchronisation method, which relies on hardware external to the PC, renders LSL synchronisation more susceptible to environmental noise and potential delays. It also complicates the integration of new devices and hinders robust TIE analysis, filtering, and adjustment of the obtained delays. In contrast, Paradigms 1 and 2 offer greater interaction with the LSL network and facilitate the addition of new devices. However, they exhibited higher relative uncertainty values, indicating lower precision and accuracy.

EEG and TMS studies often rely on precise timing to investigate neural processes. Inaccurate synchronisation can lead to temporal smearing of responses, potentially masking or distorting rapid neural events^25^. Many EEG analysis techniques, such as time-frequency or connectivity measures, depend on accurate timing information^19^. In TMS applications, the timing of pulse delivery relative to ongoing brain activity is critical, as it significantly impacts the effects of stimulation. Accurate synchronisation ensures that TMS pulses are delivered at the intended phase of neural oscillations^13^. Additionally, precise timing information is essential for effectively removing TMS-induced artefacts from EEG data, which is crucial for analysing neural responses to TMS^19^.

Despite the differences in results between hardware-based and non-hardware-based paradigms, all the paradigms examined in this study exhibit latency and error values below the acceptable limit for EEG applications, which range from 40 to 100 ms^38^. This suggests that any of the evaluated paradigms are suitable for use in EEG applications.

The choice of synchronisation method and the lack of standardisation in TMS-EEG studies present several challenges. This study highlights the importance of several crucial parameters that can optimise experimental outcomes when carefully considered. The paradigm choice could impact synchronisation performance, with each paradigm offering specific strengths and limitations. Paradigm 1 suits scenarios requiring low computational load and a simplified setup. In contrast, Paradigm 2 provides greater flexibility but demands higher data capacity. Paradigm 3 is ideal for clinical applications involving fewer devices for synchronisation but requires substantial data management resources.

In conclusion, selecting an appropriate synchronisation paradigm should consider the project’s specific requirements and the adopted experimental protocol. It is essential to weigh the advantages and disadvantages of each method discussed in this study to ensure they align with the study’s needs. This approach will facilitate optimised experimental outcomes and enhance TMS-EEG applications overall reliability and efficacy of TMS EEG applications.

This study has certain limitations, including the lack of comparative analysis between the proposed paradigms and hardware-based synchronisation systems provided by the manufacturers of the EEG or TMS devices. Such comparisons could enhance the robustness of the results and provide deeper insights into the performance of various synchronisation options.

Future work will focus on contributing to the documentation and standardisation of EEG-TMS synchronisation protocols. Additionally, integrating advanced signal processing techniques will enable detailed jitter analysis. At the same time, applying AI-driven approaches holds promise for automating pulse timestamp adjustments and minimising inter-pulse delays. These advancements could significantly improve synchronisation reliability in multimodal and real-time applications.

## Methods

This section of this study details the materials and tools utilised, including commercial and custom-developed applications. A bespoke app was designed for this research to facilitate the communication and synchronisation of TMS-EEG. The experimental protocol is outlined, encompassing the procedures adopted in the three distinct paradigms to explore various aspects of the research question. In addition, data processing techniques are described, and the statistical analysis methods employed to interpret the results are discussed.

The selection of the three paradigms studied in this work was motivated by the lack of standardisation and limited information on the comparison of TMS-EEG synchronisation systems, highlighting the pioneering nature of this research. These paradigms were designed to explore synchronisation strategies between TMS and EEG devices, primarily emphasising software-based methods due to their potential to integrate additional sensors into data collection protocols. Given the critical importance of minimising temporal discrepancies in pulse delivery between TMS and EEG for accurate analysis, two software-based and one hardware-based paradigm were proposed. The first software-based paradigm assesses the pulse delivery time when a signal, generated by an application, is simultaneously sent to both devices. The second software-based paradigm examines the pulse timestamp between TMS and EEG by delivering the pulse to the TMS and then retransmitting it to the EEG. The third, hardware-based paradigm evaluates the delay between the pulse directly generated by the TMS and its synchronisation with the EEG. All paradigms utilised LSL technology to analyse the timestamps of data transmission between devices, ensuring precise measurement of delays and robust synchronisation.

### EEG amplifier

The EEG amplifier system utilised in this study, the actiCHamp (Brain Products, Germany)^39^, supports simultaneous TMS-EEG applications. This modular and extensible system can digitise up to 160 EEG channels, enabling high-density EEG acquisition. It features eight auxiliary inputs for recording signals from additional sensors and supports high sampling rates of up to 100 kHz and low latency ($<1.5$ ms). Signals acquired from electrodes and sensors are amplified, digitised, and transmitted to a computer via USB for real-time display and storage. The system is compatible with the manufacturer’s EEG data acquisition and analysis software. The amplifier supports active electrode caps, which, combined with its advanced signal processing capabilities, ensure signal reliability by actively compensating for impedance changes, thereby reducing artefacts and enhancing accuracy.

Additionally, the amplifier includes a dedicated Trigger In port that accepts 8-bit TTL (Transistor-Transistor Logic) ranging from 0 to + 5 V, with a maximum current of 10 mA. This trigger input synchronises events with EEG data, receiving trigger pulses from external devices such as TMS equipment. When integrated with EEG system software, trigger input signals are recorded as markers alongside the EEG data, facilitating the synchronisation of specific events or stimuli during recording. A high sampling rate is necessary to prevent trigger information loss, as very short trigger pulses may not be captured effectively at lower rates.

### Transcranial magnetic stimulation device

In this study, we utilised the DuoMAG XT (Deymed Diagnostic, Czech Republic)^40^. This TMS system accommodates monophasic single-pulse and paired-pulse protocols, delivering a maximum energy output of 265 Joules, with an optional upgrade to 320 Joules, and features a pulse width of 290 µs. For repetitive TMS (rTMS) applications, it supports high-frequency protocols up to 35 Hz.

The system provides external triggering through TTL inputs/outputs and USB communication, enabling seamless integration with devices such as EEGs. TTL inputs support system synchronisation via BNC connectors, with an input impedance of 10k ohms and an output impedance of 200 ohms, accommodating input voltages from − 0.2 to 5.2 V. The signal output complies with CMOS and TTL standards, with logic levels set at 0.1 V for a logical zero and at least 4.5 V for a logical one. Output pulses have a minimum width of 200 $\upmu$s, requiring precise timing and high sampling rates from connected amplifiers. The USB port enables direct communication between external computers and the TMS device through a dedicated toolbox, which supports control via MATLAB. This integration allows for real-time monitoring of device status, adjustment of stimulation intensity, pulse triggering, configuration of recharge delays, and control of TTL OUT delays.

### Lab Streaming Layer

The Lab Streaming Layer (LSL) is an open-source project designed to standardise the collection of time series measurements in research experiments. LSL tools manage networking, time synchronisation, (near) real-time access, and optionally centralised data viewing and disk recording^37^.

LSL’s LabRecorder tool provides reliable streaming, synchronisation, and centralised data recording. It uses Network Time Protocol (NTP) for clock synchronisation across streams, ensuring accurate time stamping and data integrity. This protocol ensures that all data is synchronised accurately despite variable network latency.

The LSL distribution is versatile, comprising a core library and language interfaces in C, C++, Python, Java, and MATLAB, allowing researchers to work with their preferred programming languages. LSL’s approach to synchronised aggregation of concurrent data streams offers three main advantages that enhance data acquisition: (1) Facilitating multimodal data collection with heterogeneous and irregular sampling rates; (2) Enabling distributed measurement and data processing across multiple systems; (3) Streamlining both real-time and offline access to timestamped multimodal data through its companion XDF file format^31^.

While LSL boasts robust synchronisation capabilities, it is important to note its limitations. LSL cannot access incoming data until it is received by the microprocessor (CPU) or micro-controller unit (MCU) running the LSL software. This means LSL cannot estimate on-device delays within each recording device (i.e., the intervals between data signal input and its arrival in the software). To overcome this, measuring on-device delay for each acquisition stream at least once is necessary. Modelled as constant between setup changes, these delays can be accounted for and declared in the software. This limitation is inherent to multimodal neuroscience data acquisition systems without expected hardware clock availability^31^.

### Custom app

For this study, a custom application was developed using MATLAB App Designer to synchronise and control the TMS device pulses and the EEG amplifier triggers. The application aims to establish a communication link between TMS and EEG devices, enabling simultaneous command transmission to the TMS and the EEG amplifier via an Arduino connection. The TMS system toolbox facilitated external control of the TMS device, allowing the pulse intensity and period to be set through a serial port connection.

The interface between the EEG amplifier and the application was implemented using an Arduino UNO R4 board connected to the application’s serial port. Consequently, digital commands from the application were transmitted to the amplifier as digital trigger pulses via the Arduino board. Arduino-based interface offers several advantages, such as precise timing control and measurement capabilities, flexibility to integrate multiple devices and signals, and the ability to implement more complex triggering and synchronisation schemes.

The developed application features a user-friendly screen interface (Fig. 1) and a suite of tools specifically tailored for this study. In addition to establishing serial port (COM port) connections with the Arduino and TMS devices, the application integrates Lab Streaming Layer (LSL) tools to facilitate network connections and unified device management via LabRecorder.

A pulse configuration module within the application allows users to specify the pulse train parameters. Users can select pulse trains of 50, 100, 200, or 500 pulses with 1, 5, 10, or 20 Hz frequencies. These frequency options were chosen based on their common use in rTMS studies in previous research^41,42^.

At the end of each trial, LSL communication is terminated, and LabRecorder generates a file containing timestamp information for the connected devices. Additionally, an option for data processing was incorporated into the application, allowing for a comprehensive analysis of the trial results.

### Protocol

**Fig. 5:**
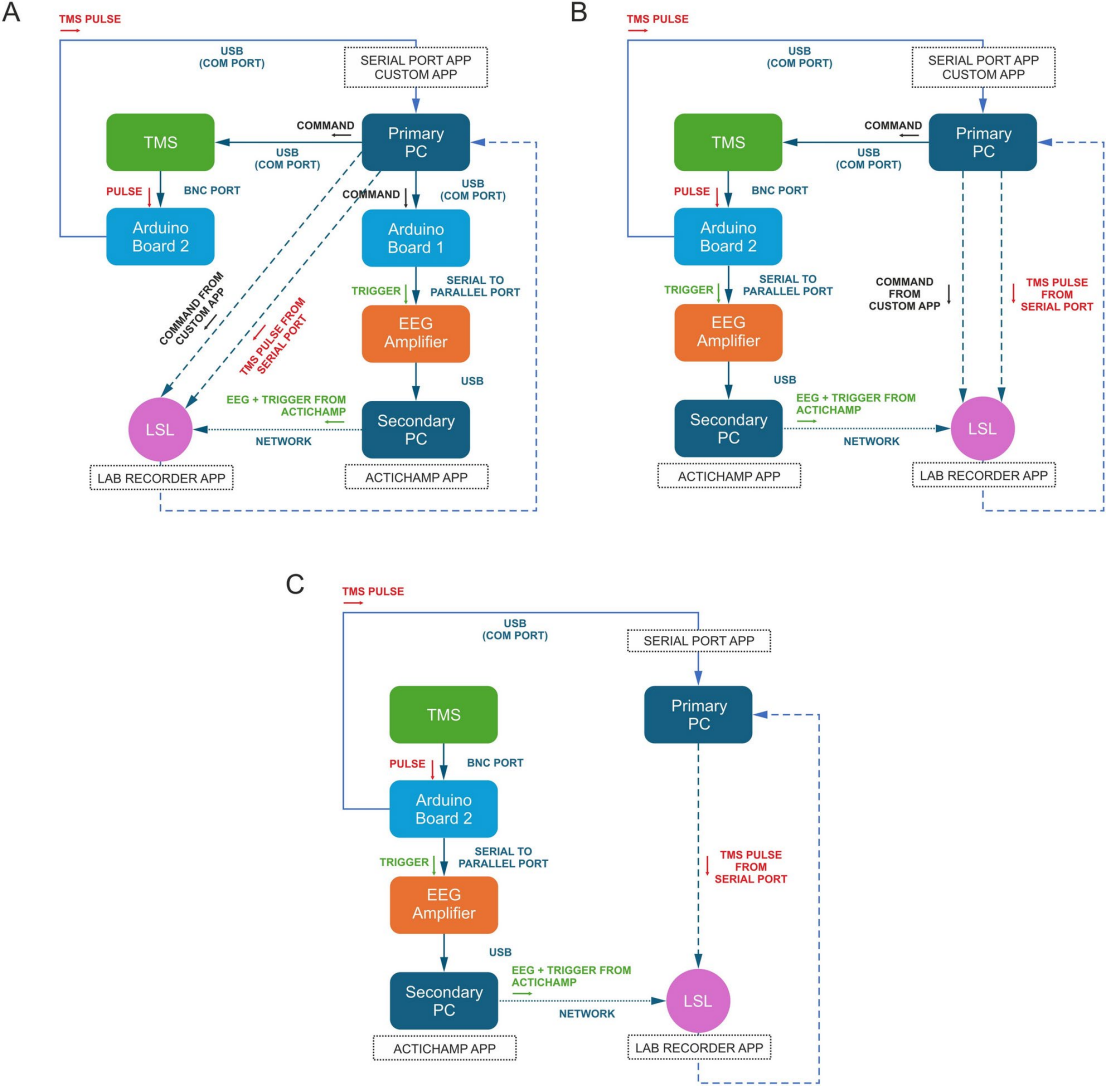
Flowchart illustrating data flow during Paradigms 1, 2, and 3. (**A**) In Paradigm 1, commands from Primary PC via the App are sent to the TMS device and Arduino board 1 through serial ports. Timestamps are captured and transmitted to the LSL network. Arduino Board 1 sends triggers to the EEG Amplifier via the parallel port. EEG signals and markers are recorded on Secondary PC using the LSL application and relayed to the LSL network. The TMS device emits an analogue pulse through the BNC TTL OUT port, measured by Arduino Board 2 and transmitted to the LSL network via the LSL Serial Port application. All devices are registered and archived by the LabRecorder application on Primary PC. (**B**) In Paradigm 2, commands from Primary PC via the App are sent to the TMS device through the serial port. Timestamps are sent to the LSL network. The TMS device emits an analogue pulse through the BNC TTL OUT port, which is transmitted to the EEG Amplifier via the parallel port. The signal is measured by Arduino Board 2 and sent to the LSL network via the LSL Serial Port application. EEG signals and markers are acquired by Secondary PC and sent to the LSL network via the application. All devices are registered, and data is stored by the LabRecorder application on Primary PC. (**C**) In Paradigm 3, commands originate from the TMS device manufacturer’s software. The TMS emits an analogue pulse through the BNC TTL OUT port, which is sent to the EEG Amplifier via the parallel port. The signal is measured by Arduino board 2 and sent to the LSL network via the LSL Serial Port application. EEG signals and markers are acquired by Secondary PC and sent to the LSL network via the application. As in the other paradigms, all devices are registered, and the LabRecorder application on Primary PC stores the data.

The study comprised three different experimental paradigms, denoted Paradigm 1, Paradigm 2, and Paradigm 3, each employing a distinct synchronisation method. The schematic representation of the three paradigms can be seen in Fig. 5. Data were recorded for each paradigm across ten trials at varying frequency values (1 Hz, 5 Hz, 10 Hz, and 20 Hz), comprising pulse trains containing 100 pulses. Utilising the LSL features in every paradigm facilitated the acquisition of timestamp information, which is crucial for synchronisation analysis. Notably, all experiments were conducted within the TMS laboratory of the Department of Psychology at the University of Liverpool and did not involve testing on human or animal subjects.

Paradigm 1, represented in Fig. 5A, entailed a synchronisation examination utilising the bespoke application described in the previous subsection developed for this study. This application concurrently dispatched commands to the TMS device and the EEG Amplifier. The experimental setup included two computers (a primary and a secondary), the TMS device, the EEG amplifier, and two Arduino boards. The primary computer executed command transmission via the application and recorded data through the LSL feature, while the secondary computer exclusively monitored EEG signals and communicated with the LSL network.

One Arduino board facilitated trigger transmission and marker recording to interface with the EEG, bridging the application and the EEG. Additionally, another Arduino board gauged the analogue electrical signal emanating from the TMS device’s BNC TTL OUT port, which was instrumental in timestamp acquisition. For precise analysis, the Arduino board’s micro-controller underwent configuration adjustments, enhancing clock settings to enable data acquisition at a 10 kHz sampling rate, ensuring faithful signal recording from the TMS, which emits pulses lasting approximately 200 $\upmu$s.

Timestamp analysis ensued from data recordings of four virtual devices present on the LSL network (Serial Port, EEG, Markers, and App) using LabRecorder. The Serial Port virtual device sends the captured electrical signals from the TMS, which are measured by the Arduino and relayed through the Serial Port connection. EEG virtual device data, encompassing 32 EEG channels and one marker channel, was recorded via the actiCHamp LSL Connector application designed for the specific EEG amplifier and provided for the LSL applications package. Marker virtual device data, signifying recorded event timestamps, was also obtained through the actiCHamp LSL Connector application. Lastly, the App virtual device in the LSL communication registered command transmission moments from the application to the TMS and EEG amplifier, established via a MATLAB script.

Paradigm 2, represented in Fig. 5B, aimed to explore an alternative synchronisation method by establishing exclusive communication between the application and the TMS, thereby circumventing intermediary communication with the EEG amplifier via the Arduino board. EEG triggers were directly recorded from the TMS’s TTL OUT port, delivering analogue pulses ranging from 0 to 4.5 V and 200 $\upmu$s in width. Utilising the LSL network, communication across four virtual devices (Serial Port, App, EEG, and Markers) persisted, with data acquisition executed by the primary computer through LabRecorder.

Paradigm 3, illustrated in Fig. 5C, sought to establish synchronisation between the EEG and TMS without the custom application of this study, employing the TMS software provided by the TMS manufacturer. The experimental configuration mirrored previous paradigms, administering pulse trains at varying frequencies. The TMS and EEG communication occurred directly via TTL OUT, with timestamp analysis conducted across three devices (Serial Port, EEG, and Markers) via the LSL network. Data acquisition remained the purview of the primary computer utilising LabRecorder.

### Data processing

The data processing involved extracting timestamp information recorded by LabRecorder, which provided files containing device-specific data, including timestamps and *timeseries*, representing signal amplitude.

To isolate the precise timing of data transmission and reception for each virtual device, relevant timestamp information was extracted. For the Serial Port and EEG data, recorded as analogical signals, this process required *timeseries* amplitude analysis. Timestamps were identified by detecting changes in the logical level of the continuous signal through a differentiation function. The exact timing of these changes was then aligned with the sample recording time to determine the corresponding timestamps.

We adjusted the timestamp offset once we obtained all devices’ timing registers. First, the virtual device that recorded the earliest timestamp was identified using an algorithm. This earliest instant of time was subtracted from all other devices to align them to the same timeline. It is important to mention that while LSL can collect unified data from devices, the tool user must synchronise it manually.

This study used time interval and latency analyses to evaluate the three synchronisation paradigms. The inter-pulse intervals (IPIs) were derived from the timestamp data for the time interval analysis. These IPIs represent the intervals between consecutive pulses recorded by the same device. Specifically, in this study, the IPIs, denoted as $IPI_d$, were calculated as the differences between consecutive pulse timestamps, $ts$, recorded by the same virtual device, as shown in Eq. (1).

1 $$\begin{aligned} IPI_d = ts(t)-ts(t-1) \quad \qquad where: \; t = 0,1,\ldots ,100. \end{aligned}$$

From the IPIs, the time interval error (TIE) was determined, which quantifies the time deviation of a clock edge from its ideal position relative to a reference point. TIE represents the accumulated error between the actual timestamp and the ideal timestamp over time, illustrating how timing errors accumulate or vary across multiple clock cycles. It serves as the discrete time domain representation of phase noise and is typically expressed in seconds^43^. This measure is particularly useful for examining the behaviour of transmitted data streams. Mathematically, TIE is defined as the difference between the recorded and expected pulse timestamps. The device’s TIE $j_d$ of each virtual device, estimated according to Eq. (2), depends on pulse train frequency *f* and the IPI.

2 $$\begin{aligned} TIE_d = IPI_d - 1/f \quad \qquad where: \; f = 1,5, 10 \; or \; 20\,Hz. \end{aligned}$$

**Fig. 6:**
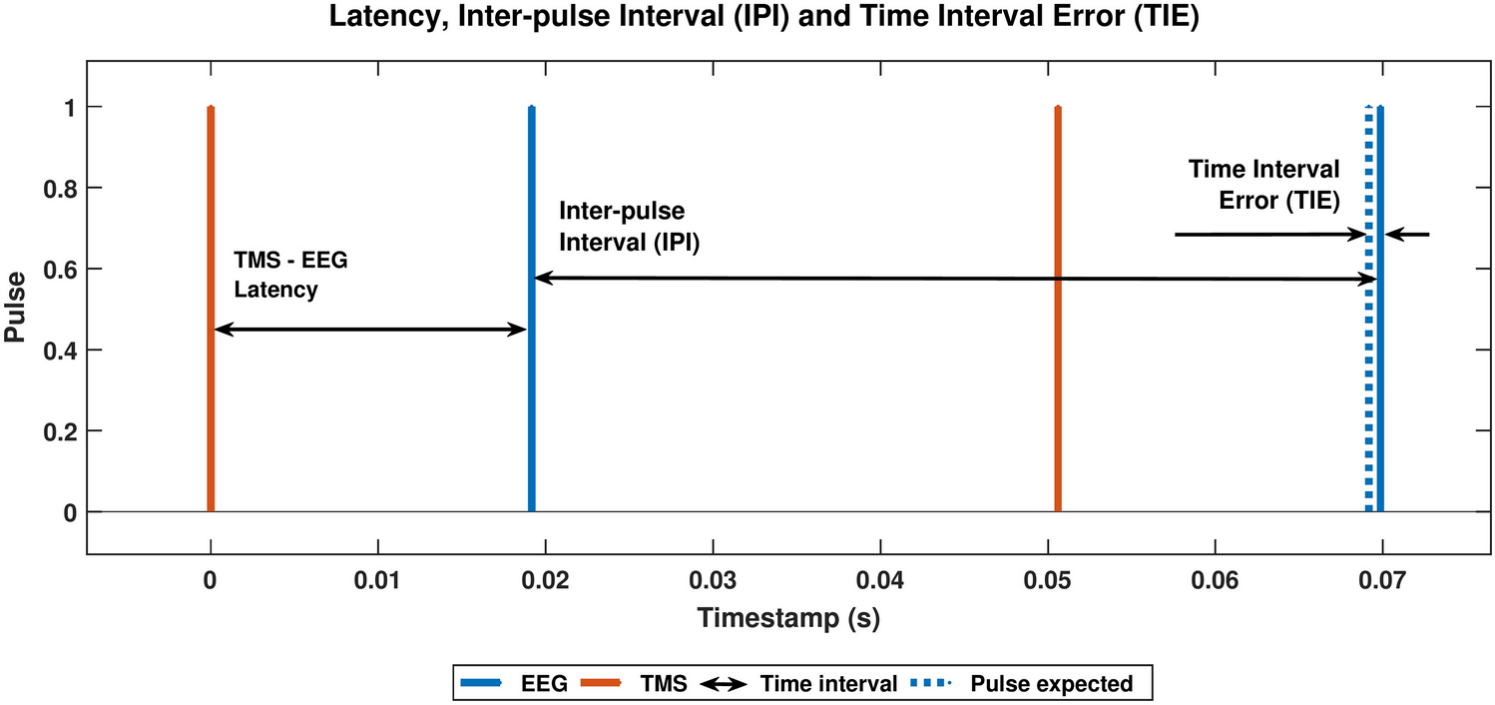
Graphical representation of Latency, inter-pulse interval (IPI), and time interval error (TIE) obtained from pulse analysis. The graph displays four pulses: two recorded by the TMS virtual device (red) and two by the EEG virtual device (blue). The TMS-EEG latency is indicated by an arrow between the TMS pulse (red) and the corresponding EEG pulse (blue), determined by the difference in timestamps between the two devices. IPI is shown by an arrow between two consecutive pulses recorded by the EEG device (blue), calculated as the time difference between these pulses. TIE is represented by the difference between the actual timestamp of a recorded EEG pulse (solid blue line) and the expected timestamp based on the pulse train frequency (dashed blue line).

The latency analysis consists of the time delay or time it takes for data to travel from one point to another. For this study, timestamps between devices were subtracted to obtain latency data, i.e., was calculated as the difference in timestamps between App and EEG, TMS and EEG, and App and TMS. This analysis aims to help understand the time between time records for the same pulse on different devices and provide insights into synchronisation. The graphical representation of IPI, TIE and latency can be seen in Fig. 6.

Furthermore, data processing involved analysing intervals and latency through both descriptive statistics and hypothesis testing, with attention to the various paradigms, devices, and frequencies employed. The descriptive statistics encompassed the following parameters: mean ± standard deviation, relative error, and relative uncertainty. The relative error, as defined by Eq. (3), quantifies the accuracy of a measurement relative to the true value. It is defined as the degree of agreement between a measured value and the true or accepted value, with a smaller relative error indicating greater accuracy.

3 $$\begin{aligned} relative \; error = [(measured \; value - expected \; value)/expected \; value] times 100 \; (\%). \end{aligned}$$

This study assessed the accuracy of timestamp recordings between pulses by analysing the relative error of IPIs. Specifically, we evaluated how closely the recorded time data matched the pulse train periods defined by the setup frequency. The relative error for IPI was calculated using Eq. (4).

4 $$\begin{aligned} interpulse \; interval \; relative \; error = (TIE_d/f_d) \times 100 \; (\%). \end{aligned}$$

The relative uncertainty, defined by Eq. (5), is directly related to the precision of a measurement. A smaller relative uncertainty indicates higher precision. The precision is quantified by the degree of agreement among independent measurements of the same quantity and reflects the reliability or repeatability of the result.

5 $$\begin{aligned} relative \; uncertainty = (uncertainty/measured \; value) \times 100 \; (\%). \end{aligned}$$

Both accuracy and precision are critical for evaluating the proper functioning of the synchronisation system, which plays a crucial role in verifying the efficiency of network services in telecommunication systems, for example^44^.

Tables, box plots, histograms, and normal curves were created to enhance the visualisation of the descriptive statistical results. Statistical hypothesis testing was performed to evaluate the significance of the comparative analyses using the Statistical Toolbox of MATLAB version 2023b. Initially, the Kolmogorov–Smirnov test was employed to assess the data distribution and determine whether it followed a normal distribution. Following the confirmation of a non-normal distribution, the non-parametric Kruskal–Wallis test was applied to analyse variance among the groups. The null hypothesis (H0) asserted that there were no significant differences between the variations, while the alternative hypothesis (H1) proposed that significant differences were present. All tests were conducted with a 95% confidence level. The Additional Information section shows a table displaying the *p-values* obtained from the hypothesis tests.

## Supplementary Information

Supplementary Information.

## Data Availability

Figures and tables derived from the hypothesis statistical analysis, which form the basis of this study’s results, are available in the Additional Information section. The raw datasets used and analysed during this study are available from the corresponding author upon reasonable request.
