# Supplementary material for: A comparative study to assess synchronisation methods for combined simultaneous EEG and TMS acquisition
Source: Sci Rep. 2025 Apr 14;15:12816. doi: 10.1038/s41598-025-97225-7 (PMC11997080; doi:10.1038/s41598-025-97225-7)
Supplement: Supplementary file 1 — Supplementary Information. [file 41598_2025_97225_MOESM1_ESM.pdf]

Additional information

The following section presents supporting data on the mean and standard deviation values for TIE and Latency. Tables I and II display the mean values for each test conducted, organised by frequency configuration and paradigm for the virtual devices analysed. These tables support the values presented in the Figures provided in the main text.

In addition, this section also provides additional information on the statistical analyses conducted in this study. The tables display the *p-values* obtained from the Kruskal-Wallis statistical test, a non-parametric test used for variance analysis. In this study, Time Interval Error (TIE) and latencies among different paradigms, virtual devices, and frequency setups were analysed to determine if the variance behaviour supported the null hypothesis (H_0) or the alternative hypothesis (H_1). H_0 posited no significant differences between the variations, while H_1 suggested that significant differences were present. All tests were conducted with a 95% confidence level.

Table III shows the inter-paradigm analyses of TIE values, Table IV presents the inter-virtual device analyses of TIE values, and Table V details the inter-frequency setup analyses for TIE values. Similarly, Table VI displays the inter-paradigm analyses for latency results, Table VII outlines the inter-virtual device analyses for latency, and Table VIII provides the inter-frequency setup analyses for latency values. Additionally, to clarify comparisons, multi-comparison graphs for each analysis (TIE values, Figure II, and latency values, Figure I) are included. The results are demonstrated in mean ranks format, where lines that cross indicate non-significant differences, and lines that do not cross indicate significant differences. The greater the distance between the lines, the greater the difference between the compared values. All tests were conducted using the Statistics Toolbox in the 2023b version of Matlab.

Virtual Device	Paradigm	Frequency	TIE Mean ± Standard Deviation (ms)
TMS	Paradigm 1	1Hz	9.726 ± 1.156
		5Hz	0.612 ± 0.750
		10Hz	0.565 ± 0.663
		20Hz	0.539 ± 0.772
	Paradigm 2	1Hz	9.977 ± 1.438
		5Hz	0.525 ± 0.925
		10Hz	0.553 ± 0.835
		20Hz	0.545 ± 0.735
	Paradigm 3	1Hz	0.131 ± 0.013
		5Hz	0.026 ± 0.015
		10Hz	0.013 ± 0.012
		20Hz	0.073 ± 0.011
EEG	Paradigm 1	1Hz	9.724 ± 1.248
		5Hz	0.610 ± 0.829
		10Hz	0.564 ± 0.761
		20Hz	0.537 ± 0.889
	Paradigm 2	1Hz	9.954 ± 1.440
		5Hz	0.525 ± 0.926
		10Hz	0.553 ± 0.834
		20Hz	0.545 ± 0.735
	Paradigm 3	1Hz	0.131 ± 0.011
		5Hz	0.026 ± 0.011
		10Hz	0.013 ± 0.011
		20Hz	0.074 ± 0.015
App	Paradigm 1	1Hz	9.733 ± 2.528E-10
		5Hz	0.621 ± 3.609E-10
		10Hz	0.586 ± 4.047E-10
		20Hz	0.566 ± 4.674E-10
	Paradigm 2	1Hz	10.028 ± 5.840E-11
		5Hz	0.583 ± 1.419E-10
		10Hz	0.591 ± 1.222E-10
		20Hz	0.578 ± 1.260E-10

Table I. Time Interval Mean Value

Latency	Paradigm	Frequency	Latency Mean ± Standard Deviation (ms)
TMS-EEG	Paradigm 1	1Hz	19.195 ± 0.167
		5Hz	19.166 ± 0.137
		10Hz	19.044 ± 0.141
		20Hz	18.878 ± 0.132
	Paradigm 2	1Hz	11.960 ± 0.013
		5Hz	11.949 ± 0.017
		10Hz	11.954 ± 0.014
		20Hz	11.925 ± 0.014
	Paradigm 3	1Hz	9.594 ± 0.010
		5Hz	9.587 ± 0.010
		10Hz	9.614 ± 0.011
		20Hz	9.590 ± 0.009
App-TMS	Paradigm 1	1Hz	8.315 ± 1.560
		5Hz	5.641 ± 0.682
		10Hz	5.502 ± 0.571
		20Hz	5.008 ± 0.663
	Paradigm 2	1Hz	8.266 ± 1.618
		5Hz	5.887 ± 0.968
		10Hz	5.728 ± 0.749
		20Hz	5.237 ± 0.913
App-EEG	Paradigm 1	1Hz	25.160 ± 2.196
		5Hz	24.749 ± 0.773
		10Hz	24.545 ± 0.664
		20Hz	23.885 ± 0.755
	Paradigm 2	1Hz	18.062 ± 1.837
		5Hz	17.836 ± 0.968
		10Hz	17.682 ± 0.749
		20Hz	17.162 ± 0.914

Table II. Latency Mean Value

Kruskal-Wallis Statistical Test		
TIE analysis - inter-paradigms		
Virtual device	Paradigm comparison	*p-value*
TMS	Paradigm 1 x Paradigm 2 x Paradigm 3	7.0599E-87
	Paradigm 1 x Paradigm 2	0.8663
	Paradigm 1 x Paradigm 3	5.8844E-78
	Paradigm 2 x Paradigm 3	1.2532E-61
EEG	Paradigm 1 x Paradigm 2 x Paradigm 3	9.5182E-86
	Paradigm 1 x Paradigm 2	8.8880E-01
	Paradigm 1 x Paradigm 3	1.2128E-70
	Paradigm 2 x Paradigm 3	4.7835E-61
App	Paradigm 1 x Paradigm 2	1.0000

Table III. *p-values* from the Kruskal-Wallis statistical test by comparing TIE values across inter-paradigms.

Kruskal-Wallis Statistical Test		
TIE analysis - inter-virtual devices		
Paradigm	Virtual devices comparison	*p-value*
Paradigm 1	TMS x EEG	0.9663
	TMS x App	0.7819
	EEG x App	0.4793
Paradigm 2	TMS x EEG	0.9725
	TMS x App	0.3724
	EEG x App	0.4063
Paradigm 3	TMS x EEG	1.0000

Table IV. *p-values* from the Kruskal-Wallis statistical test by comparing TIE values across inter-virtual devices.

Kruskal-Wallis Statistical Test		
TIE analysis - inter-frequency setups		
Paradigm 1		
Virtual device	Frequency setup comparison	*p-value*
TMS	1Hz x 5Hz x 10Hz x 20Hz	5.8833E-48
	5Hz x 10Hz x 20Hz	0.7818
	10Hz x 20Hz	0.8359
EEG	1Hz x 5Hz x 10Hz x 20Hz	6.2127E-48
	5Hz x 10Hz x 20Hz	0.8616
	10Hz x 20Hz	0.8012
App	1Hz x 5Hz x 10Hz x 20Hz	3.8031E-80
	5Hz x 10Hz x 20Hz	4.5073E-58
	10Hz x 20Hz	3.6187E-34
Paradigm 2		
Virtual device	Frequency setup comparison	*p-value*
TMS	1Hz x 5Hz x 10Hz x 20Hz	1.0152E-47
	5Hz x 10Hz x 20Hz	0.8986
	10Hz x 20Hz	0.6383
EEG	1Hz x 5Hz x 10Hz x 20Hz	1.0160E-47
	5Hz x 10Hz x 20Hz	0.8999
	10Hz x 20Hz	0.6330
App	1Hz x 5Hz x 10Hz x 20Hz	3.7442E-80
	5Hz x 10Hz x 20Hz	3.9001E-58
	10Hz x 20Hz	2.4559E-34
Paradigm 3		
Virtual device	Frequency setup comparison	*p-value*
TMS	1Hz x 5Hz x 10Hz x 20Hz	1.4111E-73
	5Hz x 10Hz x 20Hz	1.6907E-46
	10Hz x 20Hz	5.2242E-34
EEG	1Hz x 5Hz x 10Hz x 20Hz	2.5126E-74
	5Hz x 10Hz x 20Hz	7.8586E-48
	10Hz x 20Hz	5.2782E-34

Table V. *p-values* from the Kruskal-Wallis statistical test by comparing TIE values across inter-frequency setups.

Kruskal-Wallis Statistical Test		
Latency analysis - inter-paradigms		
Latency analysis	Paradigm comparison	*p-value*
TMS-EEG	Paradigm 1 x Paradigm 2 x Paradigm 3	1.8335E-232
	Paradigm 1 x Paradigm 2	1.5367E-146
	Paradigm 1 x Paradigm 3	7.8111E-147
	Paradigm 2 x Paradigm 3	1.5415E-144
TMS-App	Paradigm 1 x Paradigm 2	2.9648E-86
App-EEG	Paradigm 1 x Paradigm 2	2.0877E-130

Table VI. *p-values* from the Kruskal-Wallis statistical test by comparing latency values across inter-paradigms

Kruskal-Wallis Statistical Test		
Latency analysis - inter-virtual devices		
Paradigm	Latency analysis comparison	*p-value*
Paradigm 1	TMS-EEG x TMS-App x App-EEG	9.936E-234
	TMS-EEG x TMS-App	8.6568E-149
	TMS-EEG x App-EEG	1.1066E-140
	TMS-App x App-EEG	1.2308E-140
Paradigm 2	TMS-EEG x TMS-App x App-EEG	2.2445E-231
	TMS-EEG x TMS-App	2.6268E-144
	TMS-EEG x App-EEG	1.3040E-137
	TMS-App x App-EEG	1.5852E-138

Table VII. *p-values* from the Kruskal-Wallis statistical test by comparing latency values across inter-virtual devices

Kruskal-Wallis Statistical Test		
Latency analysis - inter-frequency setups		
Paradigm 1		
Latency analysis	Frequency setup comparison	*p-value*
TMS-EEG	1Hz x 5Hz x 10Hz x 20Hz	2.8599E-47
	5Hz x 10Hz x 20Hz	9.0816E-35
	10Hz x 20Hz	2.6047E-22
TMS-App	1Hz x 5Hz x 10Hz x 20Hz	4.3800E-47
	5Hz x 10Hz x 20Hz	2.1564E-14
	10Hz x 20Hz	4.3317E-19
App-EEG	1Hz x 5Hz x 10Hz x 20Hz	1.0347E-14
	5Hz x 10Hz x 20Hz	1.7532E-19
	10Hz x 20Hz	4.9600E-14
Paradigm 2		
Virtual device	Frequency setup comparison	*p-value*
TMS-EEG	1Hz x 5Hz x 10Hz x 20Hz	4.4233E-38
	5Hz x 10Hz x 20Hz	1.2364E-28
	10Hz x 20Hz	2.0922E-25
TMS-App	1Hz x 5Hz x 10Hz x 20Hz	8.1614E-42
	5Hz x 10Hz x 20Hz	8.4711E-12
	10Hz x 20Hz	6.3248E-9
App-EEG	1Hz x 5Hz x 10Hz x 20Hz	1.2353E-11
	5Hz x 10Hz x 20Hz	1.4357E-12
	10Hz x 20Hz	1.1367E-9
Paradigm 3		
Virtual device	Frequency setup comparison	*p-value*
TMS-EEG	1Hz x 5Hz x 10Hz x 20Hz	2.9501E-39
	5Hz x 10Hz x 20Hz	6.1815E-37
	10Hz x 20Hz	1.2847E-28

Table VIII. *p-values* from the Kruskal-Wallis statistical test by comparing latency values across inter-frequency setups

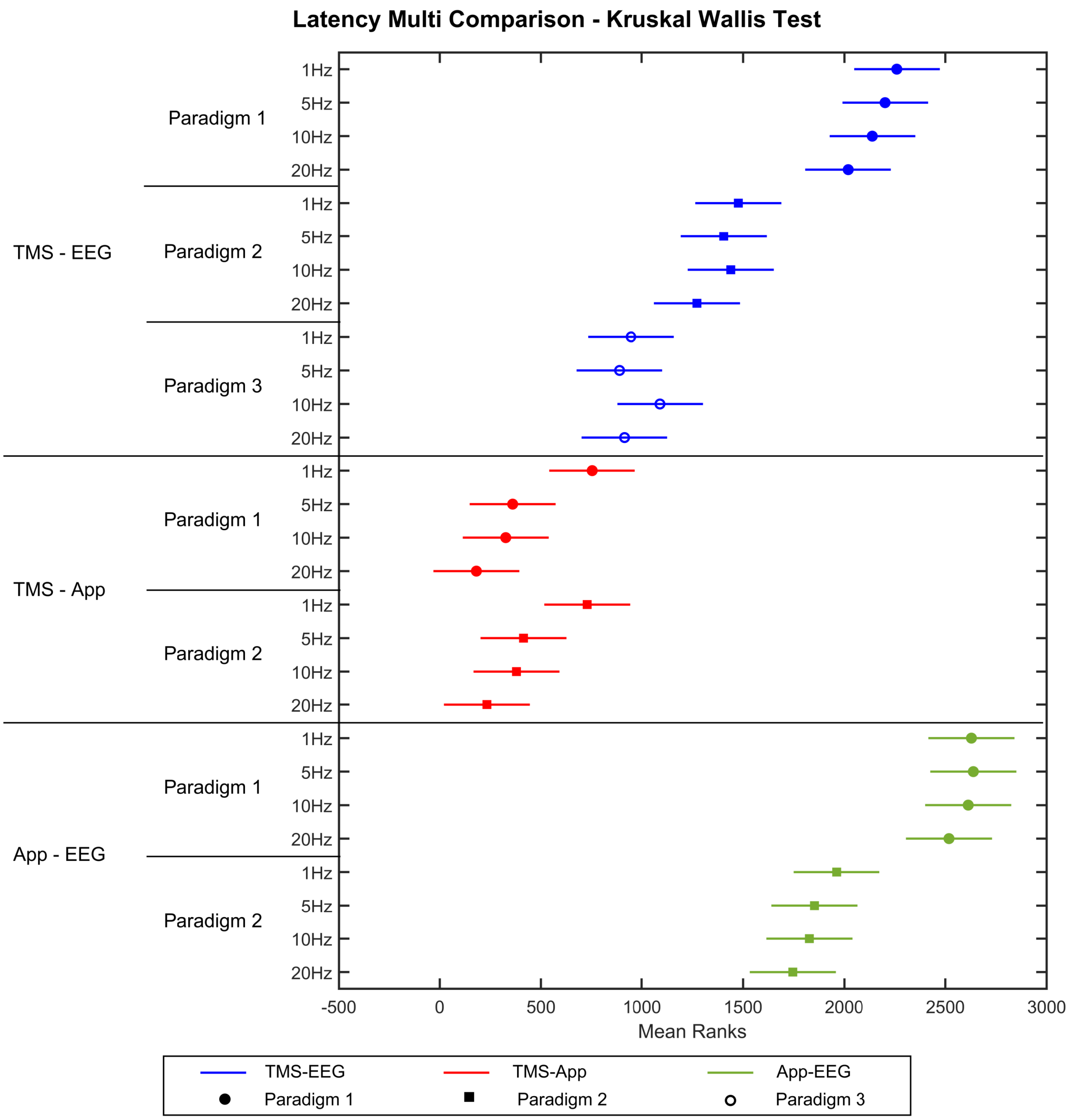

Figure I. Multi-comparison graph for latency values analyses. The graph displays mean ranks for comparisons conducted using the Kruskal-Wallis statistical test. Lines that cross indicate non-significant differences between groups, while lines that do not cross indicate significant differences. Greater distances between lines correspond to greater differences between the compared values. These visualisations clarify the significance of differences among paradigms, virtual devices, and frequency setups. All analyses were performed with a 95% confidence level using the Statistics Toolbox in the 2023b version of Matlab. A legend below the graph provides details about each element. In the graph, the blue line represents the TMS-EEG latency analysis, the red line represents the TMS-App latency analysis, and the green line represents the App-EEG latency analysis. The symbols denote the paradigms analysed: the filled circle refers to Paradigm 1, the filled square refers to Paradigm 2, and the unfilled circle refers to Paradigm 3. Frequency setup indications are found on the y-axis.

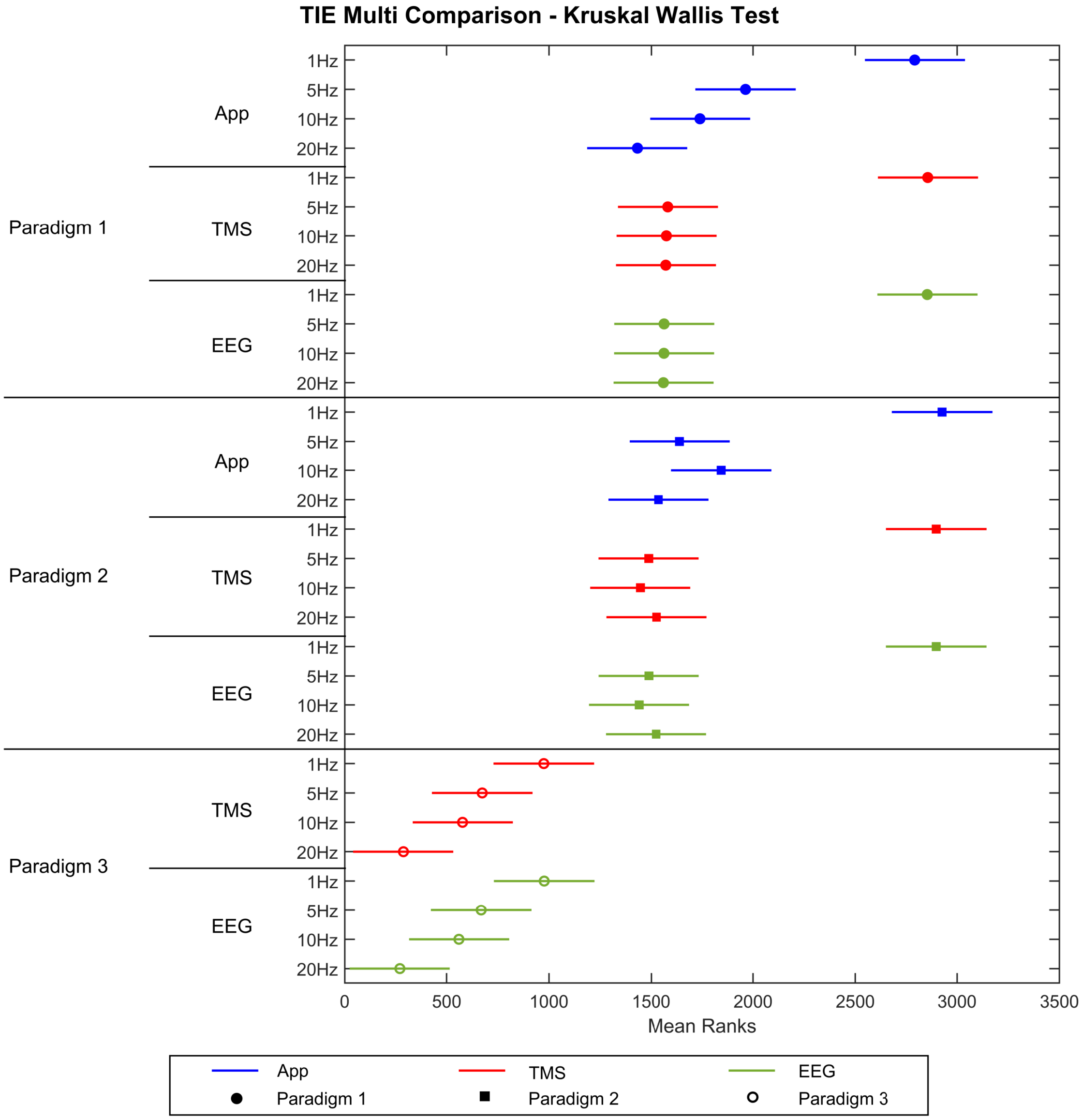

Figure II. Multi-comparison graph for TIE values analyses. The graph displays mean ranks for the comparisons conducted using the Kruskal-Wallis statistical test. Lines that cross indicate non-significant differences between the groups, while lines that do not cross indicate significant differences. The greater the distance between the lines, the greater the difference between the compared values. These visualisations help clarify the significance of differences among paradigms, virtual devices, and frequency setups. All analyses were performed with a 95% confidence level using the Statistics Toolbox in the 2023b version of Matlab. A legend is provided below the graph with information about each element present in the graph. In the graph above, the blue line represents tests conducted using the virtual device App, the red line represents the virtual device TMS, and the green line represents the virtual device EEG. The symbols denote the paradigms analysed: the filled circle refers to Paradigm 1, the filled square refers to Paradigm 2, and the unfilled circle refers to Paradigm 3. The frequency setup indications are found on the y-axis.
